# Violence against Primary Health Care Workers in Serbia and Measures for Ensuring Safe Work Environment

**Published:** 2019-12

**Authors:** Zorica J. TERZIC-SUPIC, Marina B. FISEKOVIC-KREMIC, Jovana TODOROVIC, Milena M. SANTRIC-MILICEVIC, Dejan M. NESIC, Goran Z. TRAJKOVIC

**Affiliations:** 1. Institute of Social Medicine, Faculty of Medicine, University of Belgrade, Belgrade, Serbia; 2. Primary Health Center New Belgrade, Belgrade, Serbia; 3. Institute of Medical Physiology, Faculty of Medicine, University of Belgrade, Belgrade, Serbia; 4. Institute of Medical Statistics and Informatics, School of Medicine, University of Belgrade, Belgrade, Serbia

## Dear Editor-in-Chief

Healthcare workers experience the most of non-fatal workplace violence (WPV) compared to other professional groups (1). In Serbia, in recent years, a number of healthcare workers have been attacked by patients, while two health care workers have been murdered. The likelihood of experiencing workplace violence is associated with person’s characteristics (age, gender, less job experience), occupation, circumstances, situation in which they perform their tasks, characteristics of work environments such as working alone, working late at night, poor lighting, absence of surveillance cameras, individual control of access and exit alarm system, working in unsecured areas, work load, job-related stress, and lack of supervisor support (2–4). Healthcare workers can be exposed to multiple forms of violence, in Slovenia, 25.3% of nurses were exposed to only one form of workplace violence, while 36.3% experienced two or more forms of violence (5).

To the best of our knowledge, no studies done so far have examined factors associated with experiencing two or more forms of violence. The aim of this study was to estimate prevalence and factors associated with exposure to two of more forms of violence in the workplace.

This cross-sectional study was conducted in five PHC centres in Belgrade between October 2012 and July 2013. The final sample included employees who worked in the morning shift (1757 medical and non-medical staff). The response rate was 86.8% (1526/1757). The data were collected using ILO/ICN/WHO/PSI questionnaire (6). Pearson chi-square test was used to assess differences between two groups of employees: group which had experienced only one form of violence and a group which had experienced two or more forms of violence. Experience of two or more forms of violence was the dependent variable in multivariate logistic regression model. Details of the study were described elsewhere (4).

A total of 803 (52.6%) respondents reported WPV. The prevalence of exposure to one form of violence was 27.2%, and to two or more forms of violence was 25.4%. Univariate analysis showed that exposure to two or more forms of violence was associated with work in shifts, between 6 p.m. and 7 a.m., witnessing incidents of workplace violence, work with a high number of staff in the same work setting, being member of the minority ethnic group at work, being in interaction with patients, being moved to a current place of work, and encouraged to report work-place violence. The association was found with workplace characteristics: surrounding characteristics, patient screening, patient protocols, special equipment or clothing, investment in human resource development, restricted public access, check-in procedures for staff, changing shifts, and reduced periods of working alone. The logistic regression model showed that reduced periods of working alone, changing shifts, and being the witness of incidents of WPV were associated with exposure to two or more forms of violence compared with those who experienced one form of violence. Being encouraged to report WPV, being moved to the current place of work, patient protocols, patient screening, smaller number of staff in the same work setting, and investing in human resource development, were significant predictors of lower likelihood for exposure to two or more forms of violence (Table 1).

**Table 1:** Association of socio-demographic characteristics, characteristics of work environment and measures for safe work environment with a two or more forms of violence

***Independent variables***	***OR (95% CI)***	**P*-value***
Work between 6 p.m. and 7 a.m.	1.25 (0.91–1.71)	0.176
Being the witness of incidents of workplace violence	2.33 (1.68–3.22)	<0.001
Being moved to a current place of work	0.69 (0.50–0.96)	0.028
Self sighting as a member of minority ethnic group of workplace	1.64 (0.96–2.78)	0.070
The number of staff in the same work setting (≤ 20 vs. >20)	0.49 (0.32–0.73)	<0.001
Being encouraged to report workplace violence	0.71 (0.52–0.96)	0.027
Restriction to pay fee for services	0.37 (0.12–1.17)	0.090
Patient screening	0.50 (0.30–0.83)	0.008
Patient protocols	0.55 (0.32–0.97)	0.039
Changing shifts or rotas	3.50 (2.10–5.82)	<0.001
Reduced periods of working alone	9.33 (1.96–44.30)	0.005
Investing in human resource development	0.37 (0.14–0.98)	0.045

Employees in the primary healthcare centres in Belgrade are often exposed to two or more forms of violence. The PHCs centres need patient screening and protocols, investment in human resource development, and support mechanisms for encouragement to report WPV.
